# Priapism at Diagnosis of Pediatric Chronic Myeloid Leukemia: Data Derived from a Large Cohort of Children and Teenagers and a Narrative Review on Priapism Management

**DOI:** 10.3390/jcm12144776

**Published:** 2023-07-19

**Authors:** Meinolf Suttorp, Stephanie Sembill, Krzysztof Kalwak, Markus Metzler, Frederic Millot

**Affiliations:** 1 Pediatric Hematology and Oncology, Medical Faculty, TU Dresden, 01307 Dresden, Germany; 2 Pediatric Oncology and Hematology, Department of Pediatrics and Adolescent Medicine, University Hospital Erlangen, 91054 Erlangen, Germany; stephanie.sembill@uk-erlangen.de (S.S.); markus.metzler@uk-erlangen.de (M.M.); 3 Comprehensive Cancer Center Erlangen-EMN (CCC ER-EMN), 91054 Erlangen, Germany; 4 Supraregional Center of Pediatric Oncology “Cape of Hope”, Wroclaw Medical University, 50-556 Wroclaw, Poland; krzysztof.kalwak@gmail.com; 5 Inserm CIC 1402, University Hospital of Poitiers, 86000 Poitiers, France; f.millot@chu-poitiers.fr

**Keywords:** chronic myeloid leukemia, pediatric CML, priapism, leukostasis, hyperleukocytosis, leukapheresis, exchange transfusion, pediatric CML registry, penile puncture

## Abstract

Pediatric chronic myeloid leukemia (CML) is a very rare malignancy (age-related incidence 0.1/100,000) typically presenting with leucocyte counts >100,000/µL. However, clinical signs of leukostasis are observed at diagnosis in only approximately 10% of all cases and among these, priapism is infrequent. Here, we analyze data from pediatric CML registries on the occurrence of priapism heralding diagnosis of CML in 16/491 (3.2%) boys (median age 13.5 years, range 4–18) with pediatric CML. In the cohort investigated, duration of priapism resulting in a diagnosis of CML was not reported in 5 patients, and in the remaining 11 patients, occurred as stuttering priapism over 3 months (*n* = 1), over 6 weeks (*n* = 1), over 1–2 weeks (*n* = 2), over several days (*n* = 2), or 24 h (*n* = 1), while the remaining 4 boys reported continuous erection lasting over 11–12 h. All patients exhibited splenomegaly and massive leukocytosis (median WBC 470,000/µL, range 236,700–899,000). Interventions to treat priapism were unknown in 5 patients, and in the remaining cohort, comprised intravenous fluids ± heparin (*n* = 2), penile puncture (*n* = 5) ± injection of sympathomimetics (*n* = 4) ± intracavernous shunt operation (*n* = 1) paralleled by leukocyte-reductive measures. Management without penile puncture by leukapheresis or exchange transfusion was performed in 3 boys. In total, 7 out 15 (47%) long-term survivors (median age 20 years, range 19–25) responded to a questionnaire. All had maintained full erectile function; however, 5/7 had presented with stuttering priapism while in the remaining 2 patients priapism had lasted <12 h until intervention. At its extreme, low-flow priapism lasting for longer than 24 h may result in partial or total impotence by erectile dysfunction. This physical disability can exert a large psychological impact on patients’ lives. In a narrative review fashion, we analyzed the literature on priapism in boys with CML which is by categorization stuttering or persisting as mostly painful, ischemic (low-flow) priapism. Details on the pathophysiology are discussed on the background of the different blood rheology of hyperleukocytosis in acute and chronic leukemias. In addition to the data collected, instructive case vignettes demonstrate the diagnostic and treatment approaches and the outcome of boys presenting with priapism. An algorithm for management of priapism in a stepwise fashion is presented. All approaches must be performed in parallel with cytoreductive treatment of leukostasis in CML which comprises leukapheresis and exchange transfusions ± cytotoxic chemotherapy.

## 1. Introduction

Chronic myeloid leukemia (CML) is a myeloproliferative disorder and accounts for 15–20% of all leukemias in adults with a constant incidence worldwide at 1.0–1.5 per 100,000 of the population [1]. It is characterized by the chromosomal translocation t(9;22 (q34;q11.2) resulting in the fusion gene *BCR::ABL1* which encodes for a fusion protein acting as a constitutively activated tyrosine kinase [2,3]. Patients present with high white cell count, normal or slightly depressed hemoglobin, normal or increased platelets, and, not uncommonly, with a massively enlarged spleen resulting from leukemic infiltration [4,5,6].

The disease is classically staged into chronic phase (CML-CP, approximately 90% of patients at diagnosis), accelerated phase (CML-AP), and blast phase (CML-BP), the latter being indistinguishable from acute leukemia. In untreated patients, progression to CML-BP occurs at a median of 3–5 years from diagnosis, with or without an intervening identifiable CML-AP [5].

The median age of onset is 40–60 years (younger in Asians and older in Caucasians) with a slight male predominance (1.4:1.0). CML is very rare in the first two decades of life with an age-related incidence of 0.1 per 100,000, or in other words, only 2–3% of all pediatric leukemia cases represent cases of childhood CML [7]. The characteristics of CML differ with age. Patients with CML-CP at pediatric and teenage age present commonly with more aggressive clinical and biological features than adults including a higher proportion exhibiting splenomegaly, a larger spleen size, higher platelets count, and massive leukocytosis [8,9,10,11,12,13]. While in adults, the median WBC is in the range of 60,000 cells per µL, it was demonstrated by data from large cohorts recorded in national and international registries that pediatric patients with CML-CP exhibit at diagnosis a median of 250,000 WBC per µL [9,13,14,15].

Thus, hyperleukocytosis, which is defined as a white blood cell count greater than 100,000/μL, is a common finding when pediatric CML is diagnosed. The term ‘leukostasis’ refers to ‘symptomatic hyperleukocytosis’ which can affect any organ system. Symptoms usually arise from involvement of the cerebral, pulmonary, retinal, acoustical, osseous, renal, digital, and penile (in males) microvasculature. The mechanisms underlying and resulting in leukostasis are poorly understood but it has been known for a long time that the size and more importantly the rigidity of the cells when passing through capillaries represent key factors [16,17]. Leukostasis is a medical emergency that needs prompt recognition and initiation of therapy to prevent intracranial hemorrhage and other acute or persisting organ failure [18,19,20]. Regarding priapism due to hyperleukocytosis (ischemic priapism, see below) and persisting over more than one day, irreversible loss of penile erectility will highly probably become a sequela [21].

**Figure 1:**
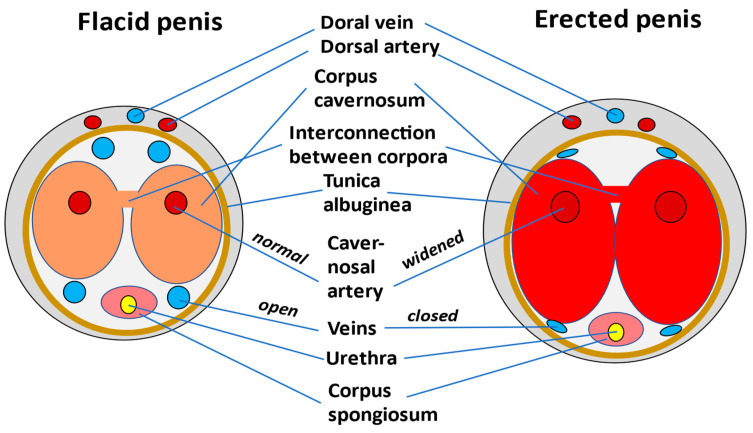
**Schematic cross-sectional diagram of the anatomy of the penis**. The smooth muscles of the corpora cavernosi and the arterial and arteriolar walls play a predominant role in the erectile process. Following sexual stimulation, these flat muscles relax causing dilatation and increased blood flow. The blood is trapped when the subtunical venular plexus are compressed between the tunica albuginea and the peripheral sinusoids. Note the interconnections between the left and right corpus cavernosum (in this schematic diagram only one interconnection is shown) which enable blood exchange in such way that only unilateral aspiration is sufficient when low flow priapism is treated (for details see text, modified from [25]).

According to the definition of the American and European Associations of Urologist, priapism is a persistent, often painful, penile erection lasting more than 4 h unrelated to sexual stimulation [22,23]. The erection is caused by the persistent engorgement of the corpora cavernosa due to disturbance of vascular mechanisms that control penile rigidity (Figure 1). Clinically and pathologically, classification separates non-ischemic (high flow, arterial) priapism, ischemic (low flow) priapism, and stuttering (recurrent) priapism. This separation is also mandatory for appropriate management in adults and children [24,25].

***Non-ischemic (high flow, arterial) priapism*** is caused by unregulated cavernous arterial blood flow. The increased arterial flow is greater than the venous outflow, resulting in an erection which is neither painful nor fully rigid and presenting with oxygenated hemoglobulin on aspiration. Non-ischemic priapism is most commonly the consequence of a trauma to the penis or perineum with an injured internal pudendal artery causing a fistula between the cavernosal artery and corpus cavernosum [26,27,28,29]. The ongoing oxygen supply makes irreversible damage or fibrosis rare. This is not a medical emergency and can be treated electively. By contrast, ***ischemic (low flow) priapism*** is a painful condition with reduced intracavernous blood flow. Pathophysiologically, the reduced venous outflow results in stasis, hypoxia, and acidosis. Etiology comprises hematological disorders, tumor infiltrate, drug-induced, or unknown (idiopathic) causes [25,30,31,32,33,34]. Ischemic priapism is the most common subtype and must be treated as an emergency. It can result in irreversible damage and fibrosis if left untreated for 24–48 h with the consequence of erectile dysfunction. Impotence has been reported in 35–90% of men with priapism lasting 5–10 days [21,35,36,37]. ***Stuttering priapism*** is a recurrent form of ischemic priapism where unwanted painful erections of >4 h duration are followed by periods of detumescence. It is observed more commonly in patients with hematological problems such as sickle cell disease, where up to one third of the affected men report attacks at night without inevitably resulting in permanent erectile dysfunction [38,39]. The goal of the management of a patient with stuttering priapism is the prevention of future episodes. Stuttering priapism may be observed in patients with CML at diagnosis but an appropriate leukoreduction in the context of the initial systemic treatment of the leukemia should manage the situation.

Penile tumescence results from relaxation of smooth muscles in the cavernosal arteries and sinusoids causing increased blood flow in both the diastolic and the systolic phases. Incoming blood is trapped by the expanding sinusoids thus causing stretching of the tunica albuginea between the inner circular and the outer longitudinal layers by which the emissary veins are occluded (Figure 1) [40]. A high resting tone of the arterial and cavernosal smooth muscles limits cavernosal arterial inflow and thus maintains penile flaccidity. Cold, sympathicotonia, or intracorporeal injection of sympathomimetics will further increase the smooth muscular resting tone [41].

Autonomic and somatic innervation of the penis via sympathetic and parasympathetic neurons located in the second to fourth sacral segments control the neuromuscular events of erection by contraction of the bulbocavernosus and ischiocavernosus muscles and penile sensation. Stimulation of neuronal and endothelial (acetylcholine mediated) nitric oxide synthase catalyzes the production of the key transmitter nitric oxide from L-arginine and oxygen. For more detailed overviews on the biochemical steering mechanisms regulating penile erection, the reader is kindly referred to reviews cited [24,25] while this article is restricted to the hemodynamics of erection [42,43,44].

After relaxation of smooth muscles in the cavernosal arteries and sinusoids, the increased blood flow rises the intracavernous pressure from 35 mm Hg to around 100 mm Hg. In contrast to the corpora cavernosa, blood flow in the glans penis and corpus spongiosum largely is maintained. There is a similar increase in arterial inflow, but as these structures lack a tunical covering ensuring venous occlusion, they act largely as an arteriovenous shunt during full erections [40].

Reflexogenic erections starting at neonatal age occur physiologically and are observed in all pre-pubertal boys. They are commonly observed during bathing, diaper changing, urethral catheterization, and with a full bladder [24,30]. Detumescence should occur within minutes after removal of the stimulus.

In CML, several factors contribute to stasis of blood flow through microvessels. As most important factor, the blood viscosity, is considered. In analogy to the hematocrit, the leukocrit is the product of leukocytes and the average volume of the individual leukocyte. Hyperleukocytosis—defined as >100,000 leukocytes per µL—will dramatically increase the leukocrit and, if exceeding 15, result in leukostasis by aggregation of blood cells in the corpora cavernosa and the penile dorsal veins might cause priapism.

When comparing pediatric leukemias, aside from CML, priapism is very rare as a complication in acute pediatric leukemias [25]. Hyperleukocytosis is observed 4-fold to 8-fold more frequently in pediatric patients with CML than with ALL or AML (Table 1). Symptoms of leukostasis may not appear in pediatric CML until the WBC count exceeds 500,000–1,000,000 leukocytes per µL [45]. One potential explanation for this finding is that about 90% of pediatric CML patients are diagnosed in CML-CP where the proportion of the most immature cells of the malignant clone—the myeloblast—is low (<5%) [16,17]. The majority of the leukocytes comprises mature segmented and banded granulocytes, and precursor cells such as promyelocytes, and myelocytes which are smaller, characterized by a low rigidity and, thus, more deformable compared to the immature myeloblasts [46,47]. Thus, propensity of blood stasis is more likely with larger, more rigid cells if present in high numbers. This is why—despite the high incidence of hyperleukocytosis—phenomena of leukostasis and associated complications are observed in pediatric CML in the range of only 10% in newly diagnosed patients which is only half of the rate reported for acute leukemias (Table 1). However, the phase of CML at diagnosis exerts an important impact. Two of the patients in this cohort presented in de novo CML-BP-lymphoid, thus harboring blasts which are indistinguishable from ALL and as rigid as in ALL.

As an additional factor promoting leukostasis, the high expression of surface molecules such as selectins and intracellular adhesion molecules (ICAMS) in cells belonging to the granulocytic developmental lineage will promote adherence to the blood vessel endothelium which may further contribute to stasis [52]. By these two rheological factors the sinusoids of the corpora cavernosa may get clogged by aggregation of leucocytes. The resulting blockage of emissary and dorsal veins hinders the venous blood return, thus causing ischemic priapism in CML [25,53].

The third additional contributing mechanical factor is splenomegaly. Compression of the abdominal veins by the enlarged spleen will obstruct the smooth venous outflow of the sinusoids in the corpora cavernosa. Splenomegaly is observed as a massive (>5 cm below the costal arch) or moderate (3–5 cm) organ enlargement in 52% and 26% pediatric patients with CML, respectively [41,54]. Also of note, lower rates of excessive splenomegaly in association with hyperleukocytosis are observed in acute leukemias when compared to CML [20].

Our understanding on priapism as an initial finding in larger series of patients with pediatric CML is sparse, mostly based on case reports only, and may also be underreported. Therefore, we retrospectively analyzed the pooled data from the worldwide registry on pediatric CML and from the national German registry CML-paed comprising 628 and 199 patients, respectively, of patients aged ≤18 years at diagnosis of CML [14,40]. Both registries already have provided valuable knowledge on specific findings when comparing adult to pediatric CML [10,55,56,57,58,59,60,61,62,63,64,65,66]. From these cohorts we here now present information on cases with priapism and their clinical features, therapy applied, and long-term outcome.

## 2. Methods

This analysis is based on the data acquired nationally (i) in Germany through studies CML-paed I and CML-paed II run as prospective, investigator-initiated, academic, multicenter, open-label, single-arm, clinical trials which subsequently merged in the year 2016 into the CML-paed Registry established in Erlangen/Germany [67,68,69] and (ii) worldwide from the prospective, investigator-initiated, academic, multicenter, open-label international I-CML-Ped Study forming the basis of the International Registry of Childhood CML at Poitiers/France [14]. Both registries are enrolling patients with CML in all phases less than 18 years of age at diagnosis.

Enrollment to the registries is ongoing and was conducted prospectively in study CML-paed I in Germany from 1994 until 2004 and thereafter in study CML-paed II from 2004 until 2015. The I-CML-PED Study initially recruited retrospectively from January 2000 until December 2010 and then prospectively from January 2011 until today. The protocols were conducted in accordance with the Declaration of Helsinki, approved by each of the responsible institutional ethical boards and registered (University Hospital of Dresden, Dresden, Germany, EK282 as of 8 December 2006, EUDRACT-2007-001339-69, www.clinicaltrials.gov NCT00445822; University Hospital of Erlangen, Erlangen Germany, EK 236-18 B; University Hospital of Poitiers, Poitiers, France, CIC09-33 PediatricCMLRegistry, EUDRACT-2010-023359-27, www.clinicaltrials.gov NCT01281735, assessed 2 May 2023].

Patients enrolled into the registries during the time period from 1/1994 to 12/2021 were analyzed. Written informed consent was obtained from the children and/or their legal guardians. Data regarding sex, age, and clinical and biological features including complications at presentation and the lines of specific CML treatment were collected from participating centers using standardized forms at diagnosis and in regular intervals thereafter. Diagnoses were confirmed by central reference review as per the current guidelines of the criteria of the European LeukemiaNet and patients <18 years old diagnosed with CML between 1 January 1994 and 31 December 2021 were eligible [70].

Patients with priapism were treated as emergency cases based on the judgement of the treating physician. When all patients had achieved adult age, data on penile functional outcome were collected via the national registries in France and Germany using a standardized paper-based questionnaire to be filled-in during follow-up visits for CML treatment in the hospital, at home, or by telephone interviews conducted by a physician.

## 3. Results

Data analysis from the registries resulted in a total of 827 children and adolescents, among them 491 males <18 years old, and identified 16 (3.3%) male patients with priapism leading to a diagnosis of CML. Characteristics of the patients can be depicted from Table 1.

**Figure 2:**
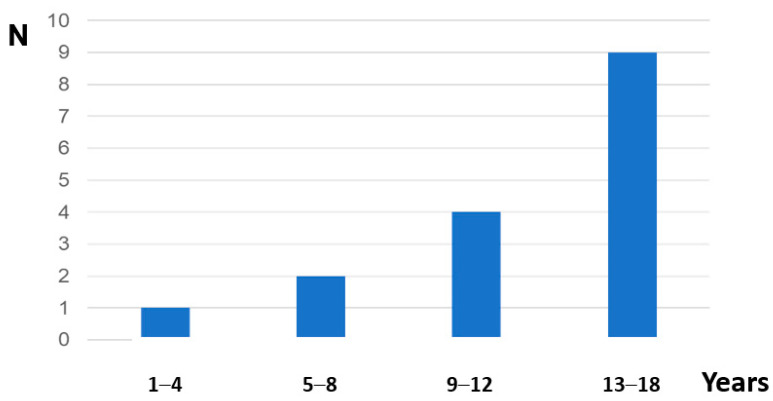
Age distribution of patients with priapism at diagnosis of pediatric CML.

The age ranged from 4 to 17 years (median 13.5 years, mean 12.3 years) with *n* = 7 boys presenting at prepubertal age (<12 years) (Figure 2). Twelve patients were in chronic phase, while two patients each were in accelerated or blastic phase of CML. All patients exhibited splenomegaly, which was moderate in five patients with a size range of 2–4 cm below the costal margin, and massive, with a size range of 6–17 cm (median 12 cm, mean 11.5 cm), in the remaining individuals.

Massive hyperleukocytosis was diagnosed in all patients with the WBC ranging from 236,700 to 899,000 cells per µL (median 470,000/µL, mean 427.575/µL). Platelet count was low in one boy diagnosed in CML-BP (Pat#1) and in the normal range (150,000 to 450,000/µL) in 10 patients, while the remaining 5 patients (Pat#2, Pat#6, Pat#7, Pat#8, Pat#12) presented with platelet counts ranging from 537,000 to 1,130,000/µL (Table 2). In all but one patient (Pat#15), a low hemoglobin value <10 g/dL was present ranging from as low as 4.9 g/dL (Pat#3) to 9.8 g/dl (Pat#16) (median 8.0 g/dL, mean 7.7 g/dL).

In all boys, the manifestation of priapism preceded the diagnosis of CML. No data on the duration of this interval were available in five patients. Stuttering priapism with episodes of spontaneous detumescence was reported by seven patients (Pat#1, Pat#3, Pat#4, Pat#8, Pat#9, Pat#10, Pat#13) lasting over a period of 24 h (Pat#1), over a period of several days to 2 weeks (Pat#4, Pat#8, Pat#9, Pat#13), over 6 to 8 weeks (Pat#3, see also case vignette #1), and maximally >3 months (Pat#10). The remaining four patients reported permanent priaptic erection over 2 to 11 h prior to hospital admission (Pat#2, Pat#5, Pat#7, Pat#12).

The interventions selected on admission to treat priapism and/or to lower the WBC during the following days covered a broad spectrum. Emergency management could not be retrieved in detail from the records due to insufficient documentation in five patients. Out of the remaining 11 patients, 2 patients received exchange transfusions which were followed by ALL induction chemotherapy in a 4-year-old boy (Pat#1) presenting in CML-BP-lymphoid and by low-dose ARA-C in a 14-year-old boy with CML-CP (Pat#10). Another four patients (Pat#4, Pat#5, Pat#8, Pat#16) were also managed without penile interventions by heparin plus ALL-induction chemotherapy (Pat#16 with CML-BP-lymphoid), or by heparin plus urokinase plus hydroxyurea (Pat#8), or by hydroxyurea plus intravenous fluids (Pat #4) or by leukapheresis plus hydroxyurea plus low-dose ARA-C (Pat#5).

Data for a detailed analysis on penile interventions were documented in nine boys (Table 2). Penile puncture to release dark colored, hypoxic blood, and flushing the corpora cavernosa with saline was performed in six patients (Pat#2, Pat#3, Pat#8, Pat#9, Pat#12, Pat#13). Due to persisting priapism, this procedure was followed by adrenaline injection in four of them (Pat#2, Pat#8, Pat#9, Pat#12) and a spongiosocavernous shunt operation in one 7-year-old boy (Pat#2). Five out of these six patients (Pat#2, Pat#3, Pat#9, Pat#12, Pat#13) also underwent 1–3 leukapheresis sessions in conjunction with chemotherapy applied in parallel in three of them with either hydroxyurea (Pat#9, Pat#12, Pat#13) or low-dose cytarabine (Pat#3).

Data on the long-term outcome of penile function were successfully collected by a standardized interview in 7 out of 15 (47%) patients (mean age 20 yrs, range 18–25 yrs) surviving after diagnosis of CML for mean 9 years (range 4–25 yrs). All seven patients (Pa#1, Pat#3, Pat#4, Pat#5, Pat#10, Pat#12, Pat#13) reported normal penile function with full erection and ejaculation (Table 2).

Regarding the outcome of CML treatment, all but two patients (Pat#8, Pat#13) survived with a median follow-up of 48 months (median 78 months, range 6–155 months). Four patients underwent SCT: two of them were diagnosed with de novo CML-BP (Pat#1, Pat#16) and one of them (Pat#11) with CML-AP while the fourth patient (Pat#8) was diagnosed in CML-CP with an extremely high platelet count above 1 million per µL. He received multiple chemotherapies followed by an unrelated cord blood transplantation which was complicated by multiple infections and death due to pneumonitis 6 months post SCT. The other fatality (Pat#13) occurred after progress to blastic phase 8 years after diagnosis. Details on the outcome of CML in the remaining 14 patients who are all alive and achieved MR3 or better are depicted in Table 2.

## 4. Discussion

### 4.1. Historical Survey on Priapism

In the Greek and Roman mythology, Priapos—the son of Aphrodite and Dionysos—was the god of fertility, vegetables, garden, and male generative power. He is depicted by his permanent oversized penile erection which gave rise to the term priapism. Priapism is already mentioned in Ebers’ ancient Egyptian papyrus (16th century BC). The Greek physician Galen of Pergamon (216–129 BC) and the medieval physician G. de Chauliac (1300–1368) both thought that the condition was due to dilatation of the arteries [71]. Traumatic causes of priapism associated with spinal cord injury also had been empirically recognized since ancient Egyptian times and comprise, e.g., the description of anatomic findings in priapism of public hanging victims by Leonardo da Vinci [71]. The first modern descriptions of non-traumatic priapism were given by the French physician T. De Héry in 1552, by the German physician H. Petraens in 1616 [72,73], and the first account appearing in the English literature by J. W. Tripe in 1845 [74]. The present pathophysiological concept of vascular stasis and reduced venous outflow (‘congestion and slowing of the blood stream’) was firstly delineated by F. Hinman in 1914 and further expanded by his son [75,76].

In patients with hyperleukocytosis due to leukemia, leukostasis by aggregation of blood cells in the corpora cavernosa and the penile dorsal veins is considered the most important factor to cause priapism. In such cases with CML, compression of intra-abdominal veins and consecutive venous congestion of the corpora cavernosa due to massive splenomegaly may contribute as an additional factor [25]. CML was first described in 1845 in autopsy cases by J.H. Bennett in Glasgow and by R. Virchow in Berlin [77,78]. This was followed by the first diagnosis of CML in a patient while still alive by H. W. Fuller in Manchester already one year thereafter [79,80]. The first report of priapism in association with CML was given by Lissauer who described a 20-year-old patient treated near the town of Kassel/Germany in 1865 (see Case Vignette #1) [81]. Already in 1879, F. Salzer from Berlin discussed a hindered blood circulation in the smaller penile vessels of the corpora cavernosa in conjunction with the formation of thrombi as a possible pathophysiological mechanism of priapism in leukemia [82].

In pediatric patients, CML is very rare and evidently priapism associated with this condition is even rarer. To the best of our knowledge the first case describing a 10-year-old Italian boy was published by G. Maciotta in 1934 [83]. This was followed by reports on a prepubertal 12-year-old boy from France by A. Depaillat in 1954 [84], and presentations of an 8-year-old boy by D. Ritz in 1964 and of a 7-year-old boy by R.G. Graw in 1969 both from the USA [85,86]. The youngest patient so far described with priapism and a diagnosis of CML is a 7-week-old baby reported by L. Graivier in 1971 presenting with hepatosplenomegaly and blastic phase of atypical CML (moderate leukocytosis of 35.000 WBC per µL, 40% lymphoblasts, no detectable Philadelphia-chromosome) [87].
**Case vignette #1** **The first case of priapism associated with CML described in the German medical literature, Lissauer 1865,** [81] [German]……betrifft einen jungen Mann von 20 Jahren aus der Nähe von Cassel [modern writing: Kassel] ….Oedem, namentlich der linken unteren Extremität … auch Zeichen von Anämie, sowie Verdauungsstörungen und Catarre waren vorhanden. Milz enorm vergrößert ….In der späteren Behandlung ….trat dann eine mehrere Tage anhaltende durch kein Mittel zu beseitigende Erection ein, …… [English, author’s translation]. …..concerning a young, 20 year-old man living near Kassel…..presenting with edema, namely of the lower left extremity …. also signs of anemia, as well as disturbed digestion and catarrh. The spleen was enlarged enormously. Later during treatment ………an erection occurred persisting for several days and not resolving by any remedy……

### 4.2. Epidemiology and Frequency of Priapism in Pediatric CML

Priapism is observed in all age groups from newborn to elderly and has an incidence of 1.5 per 100,000 [88]. While mostly seen in males with sickle cell disease, it may be the initial symptom of hyperleukocytosis in leukemias such as CML. During a 12-year period in a single tertiary center in Turkey, 71 patients presented with priapism and 24 (34%) out of these were affected with CML [89]. In a recent literature-based analysis covering the period from 1960 to 2020, E. Ali and coworkers analyzed 68 publications including case reports and case series on a total of 102 patients with priapism and CML [90]. The youngest patient was 7 weeks old, and the oldest was 60 years old. In this pooled data analysis priapism was observed more frequently in younger patients with CML. Depending on the upper age limit defining a ‘pediatric’ cohort, *n* = 41 (40%), *n* = 34 (33%), *n* = 21 (21%), and *n* = 17 (17%) patients, respectively, out of the 102 patients were children and adolescents in the age range 0–21, 0–18, 0–16, or 0–14 years, respectively. Evidently these data on pediatric CML and priapism are biased by the fact that only published reports were analyzed. A more systematic approach sourced from the CML Committee of the Japanese Pediatric Leukemia/Lymphoma Study Group. The authors report leukostasis as a complication of hyperleukocytosis in 23 out of 238 patients (9.7%) younger than 20 years when diagnosed with CML at 93 hospitals between 1996 and 2011 [59]. Priapism was seen in 4 cases (1.5%) of the total cohort. Also, a report from a trial on 40 French pediatric patients describes signs of leukostasis in 4 children (10%) and priapism in 1 boy (2.5%) only [9]. These findings—perhaps due to higher case numbers—differ considerably from a much higher reported 60% rate of leukostasis observed in 6 out of 10 pediatric CML patients described in the older literature [91].

In the, so far, largest pediatric cohort described and analyzed here, 16 out of 491 (3.2%) boys presented with priapism at diagnosis of CML, a proportion which is twice as high compared with the report from Japan. Priapism was not observed at a distinct age or in association with pubertal status but paralleled the incidence of CML which is higher in the second than in the first decade of life (Figure 2).

### 4.3. Diagnostic Approach to Priapism

Ischemic priapism is an acute emergency with treatment delay resulting in irreversible damage of corporal tissue and the ultimate consequence of erectile dysfunction [92]. Blood stasis (low flow) causes progressive local hypoxia, hypercapnia, and acidosis [30]. Priapism-related pain is a cardinal feature of ischemia due to compartment syndrome. Interstitial edema of the corpora cavernosa develops by 12 h followed by endothelial destruction of sinusoids with thrombocyte adherence after 24 h [93]. Necrosis of cavernosal tissue and fibrosis have been observed by 48 h after onset of priapism [30,92].

**Figure 3:**
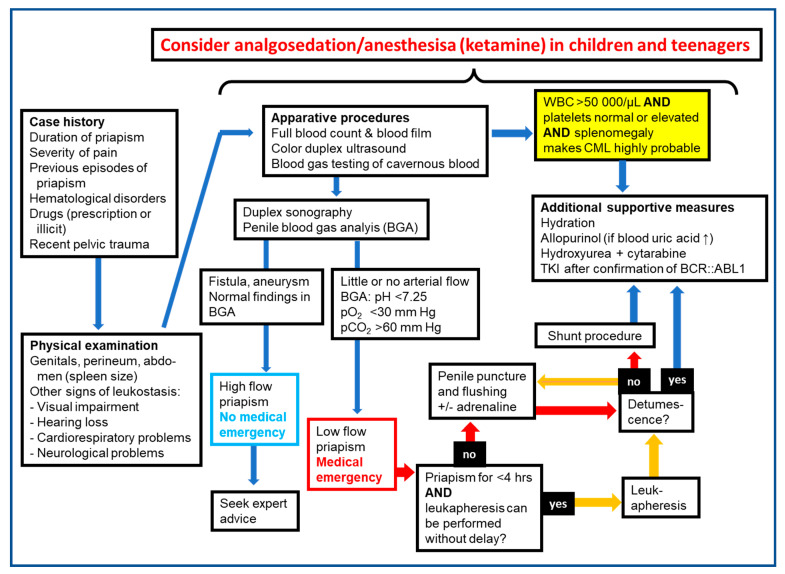
Algorithms for diagnostic procedures and therapeutic management of priapism in patients with pediatric CML (Modified from [23,24,25,96,97,98,99]). For details see text. Abbreviations: BCR::ABL1 = Breakpoint cluster region-Ableson leukemia 1 (chromosomal rearrangement representing the hall mark of CML), BGA = blood gas analysis, CML = chronic myeloid leukemia, hrs = hours, TKI = tyrosine kinase inhibitor, WBC = whole blood count.

History taking and physical examination are the first important steps when encountering cases of priapism. Assessment whether it is ischemic (low flow) or non-ischemic (high flow) in nature will determine the management pathway required (Figure 3).

Key questions when taking the history must focus on the duration of priapism (associated with the future risk of erectile dysfunction), on previous episodes of priapism (stuttering priapism), on underlying hematological disorders (e.g., sickle cell disease, which is the major cause of priapism in children), or a recent pelvic trauma (e.g., a straddle injury by a bicycle bar can cause high-flow priapism) [29,94]. Massive pain is typically associated with low-flow priapism. With a priapic penis, the patient may be writhing in pain; however, its absence is an unreliable indicator of ischemic priapism [24]. In contrast, high flow priapism never is associated with penile pain. Prescribed drugs causing priapism reported mostly in adults comprise antipsychotics (chlorpromazine, olanzapine, quetiapine, trazadone), PDE-5 inhibitors (sildenafil), anti-depressants, anti-hypertensives (including a-blockers), and depot testosterone, while illicit drugs also encountered in teenagers are cocaine, ecstasy, and marijuana [25,95].

Physical examination must not overlook any injury caused by a recent trauma to the patient’s pelvic, genital, or perineal areas possibly resulting in non-ischemic priapism. Also of note, the onset of non-ischemic priapism typically is delayed by a few days after the injury due to vessel spasm, formation of fistula, aneurysm, or a clot which is slowly resorbed [24]. Assessment of the penile rigidity may further help to discriminate the types of priapism. Unlike in a normal erection, the glans penis is typically not affected in either type of priapism and appears soft, while the corpora cavernosa are rigid in ischemic priapism compared to the less rigid state in non-ischemic priapism [25]. Concerning underlying hematological conditions, petechia due to thrombocytopenia and splenomegaly must not be overlooked (see Case Vignette #2) [41]. Besides priapism, the blood’s hyperviscosity in pediatric CML might cause visual impairment due to retinal papilledema and hemorrhages, hearing impairment, and pulmonary and CNS symptoms [52].

Among laboratory investigations, a full blood count and blood film will allow to identify underlying leukemias, sickle cell disease, and platelet disorders. A coagulation screen should identify patients with a hypercoagulable state. Urinalysis with toxicology screening may be helpful when searching for psychoactive drugs.

Penile blood aspiration (see next section) with blood gas testing and color duplex ultrasound are the two key techniques to discriminate high-flow (non-ischemic) from low-flow (ischemic priapism). Blood when aspirated from the corpus cavernosum in ischemic priapism has a dark color and blood gas testing exhibits values typical for hypoxia with low oxygen pressure, acidosis with low pH, and high carbon dioxide pressure (Figure 3). Non-ischemic values are similar to those of arterial blood (pO_2_ > 90 mmHg, pCO_2_ < 40 mmHg, pH 7.4). An animal study demonstrated that measuring glucose might aid in giving a prognosis of the reversibility of priapism as a low glucose predicts smooth muscle damage [30]. Whether this is appropriate in pediatric cases has not been investigated; however, in severe cases with a long duration of priapism, it may be considered and is easily performed if glucose values are part of the blood gas analysis tests.

Color duplex ultrasonography (CDU) is a non-invasive procedure detecting little or usually absent arterial flow through the cavernosal arteries in ischemic priapism, but high-flow in non-ischemic priapism [24]. CDU is a simple, reproducible, non-invasive, painless procedure which is helpful for guiding the management of priapism but requires expertise and may not be accessible in all emergency ambulances for 24 h per day. The up-front use of magnetic resonance imaging is time consuming, expensive, and not recommended; however, it can assess smooth muscle viability and predict erectile function restoration post-event [23,31]. Finally, elective pudenda arteriography can be diagnostic as well as therapeutic and, therefore, it should be reserved for the management of high-flow priapism when embolization is required [96,100].
**Case vignette #2** **Stuttering priapism in a 7-year-old boy with CML** Ongoingly over a period of 6–8 weeks, a 7-year-old prepubertal boy (Tanner stage 1) complained penile erections lasting for 7–8 h occurring once or twice weekly (stuttering priapism). During this period the parents had presented the boy four times to different urologic and pediatric practitioners; however, when physically examined at the appointment, penile erection had resolved. No further diagnostics was initiated with the single exception of a urologist who recommended to contact a pediatric hemato-oncologist without delay. For unknown reasons, the parents did not follow this recommendation. Abdominal palpation was either not performed by the physicians, or had overlooked or not interpreted adequately the massive hepatosplenomegaly (liver 5 cm, spleen 7 cm below the costal arch) which the boy presented at hospital admission. When another episode of priapism occurred, the boy was presented in the evening hours to the urology department at a university hospital where penile blood aspiration and irrigation without injection of sympathomimetics was performed and detumescence successfully achieved. The high WBC of 635 000/µL prompted the transfer to the department of pediatric hemato-oncology. Contact with the department of transfusion medicine was sought immediately and the apheresis equipment was transported to the pediatric intensity care unit (ICU). On the ICU following insertion of a catheter to the jugular vein, leukapheresis was initiated immediately with a flow rate of 3–5 mL per min over 4 h until midnight. A total blood volume of 2.1 L was processed (1.1-fold the calculated boy’s total blood volume of 1.9 L, bodyweight 23 kg), resulting in a decrease in the WBC to 450 000/µL. One unit of packed red cells was transfused in parallel to compensate the low Hb of 3.1 mmol/L (4.99 g/dL; Hct 14%) at presentation. No adverse events were observed. Cytarabine and hydroxyurea and allopurinol were started next morning and leukapheresis was repeated after 12 h with the intention to lower the risk of another episode of priapism. This brought down the WBC to 343/µL an no further episodes of priapism were noted. After the diagnosis of pediatric CML was confirmed by FISH karyotyping showing the Major-BCR::ABL1 rearrangement, treatment with imatinib was initiated on the third day after admission When after one year of therapy, no optimal response according to the ELN recommendation was achieved, treatment was switched to the second generation TKI dasatinib which resulted in deep molecular response (MR4.5). In an interview performed with the patient when he was 20 years old, he indicated normal erectile function.

### 4.4. Therapeutic Approach to Priapism

**Figure 4:**
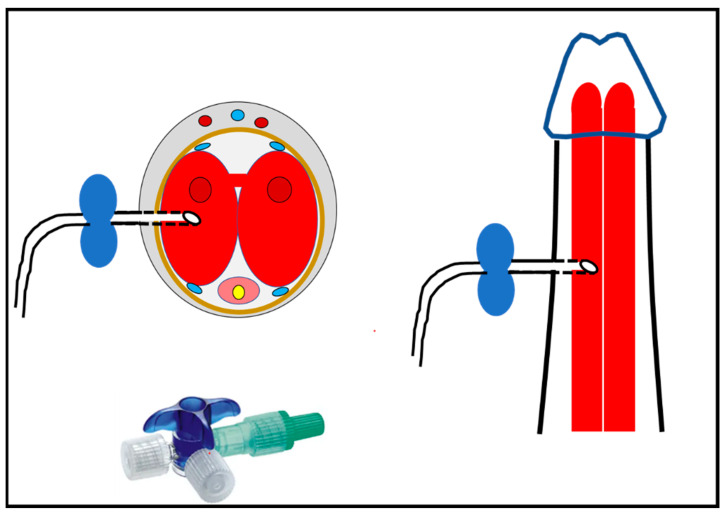
Technique of corporal blood aspiration with a butterfly needle. Insertion is performed laterally at a middle position of the shaft to avoid damaging the corpus spongiosum/urethra or the dorsal neurovascular bundles. In cases of low flow priapism, unilateral aspiration is sufficient as interconnections exist between the left and right corpus cavernosum. A three-way tap is connected to the tube of the butterfly needle allowing irrigation and aspiration (Modified from [24]).

In adults with CML and priapism, initial therapeutic aspiration of blood for blood gas testing is combined with irrigation of heparinzed saline and/or intracavernous injection of phenyl-ephrine (adrenaline). After a tourniquet is applied to the base of the penis, a butterfly needle is inserted laterally through the penile skin in the middle of the shaft avoiding the urethra or dorsal neurovascular bundle (Figure 4). The procedure is outlined in more detail elsewhere [101]. In children and teenagers with CML presenting at the typical age of 4 -18 years old, these procedures should be performed under conscious sedation and with local anesthesia (penile block) [102]. If pediatric anesthetic expertise is readily available, dissociative sedation with low-dose ketamine, propofol, fentanyl, or morphine are the drugs of choice [103]. In particular, ketamine should be used preferentially as it may resolve priapism (see Supplement 1 [104,105,106,107,108,109,110,111]).

To reduce skin bruising, passing the needle through the glans is recommended by some authors, although this may cause problems when subsequent distal shunt procedure is performed [24]. Unilateral aspiration is sufficient as the two penile corpora are interconnected. Using a three-way tap for repetitive flushing with saline allows to minimize needle passages (Figure 4). In pre-pubertal boys, a 21–23 gauge butterfly needle, and in adolescents, a bigger 19 gauge needle size is appropriate but problems may arise when clots have to be evacuated [102,112]. Aliquots of 3–5 mL of blood should be aspirated not exceeding a total volume of 30 mL (prepubertal) to 50 mL (adolescents) until the bright red color of oxygenated blood appears [24,112]. While some authors advocate repetitive aspirations with a maximum duration of one hour to achieve penile detumescence others recommend repeating irrigation only two or three times. On its own, aspiration has a success rate of approximately 30% [25]. If no permanent detumescence is achieved, immediate injection of adrenaline solution should follow [24,25,97]. In successful cases, applying pressure for several minutes after needle removal will reduce the hematoma rates [95,102,113]. Rare complications of cavernosal puncture include infection, urethral lesions, and non-ischemic priapism [24].

In the cohort described here, penile puncture was performed in 5 of 9 (55%) boys with analyzable data. Injection of sympathomimetics was required in four of the five patients. This is well in the range reported by other authors but evidently our cohort is too small for a more detailed comparison.

### 4.5. Intracavernous Sympathomimetics

If ischemic priapism persists following aspiration and irrigation with saline, intracavernous injection of sympathomimetics should be performed. Cardiovascular monitoring (blood pressure, electrocardiogram) is strongly recommended as side effects due to peripheral vasoconstriction as well as positive inotropic and chronotropic effects on the heart may be possible. Rarely acute hypertension, reflex bradycardia, tachycardia, arrhythmias, and non-cardiologic side effects such as headache or dizziness have been observed [36,98]. As recommended by the American Urological Association guideline, phenylephrine—a selective alpha-1 adrenergic agonist which lacks beta-mediated cardiac ionotropic and chronotropic effects—is the sympathomimetic agent of choice due to its lower risk of cardiovascular side effects [114,115]. Other sympathomimetic agents that can be used are adrenaline or etilefrine, while metaraminol (a pure alpha-agonist) should not be used due to its high cardiovascular complication rate [116]. There are no direct comparative studies between these agents and there is no specific guidance on dosages for pediatric patients. As a pragmatic approach, JF Donaldson and coworkers recommend selection of the sympathomimetic to be used depending on what is readily available [24]. Suggested dosages, preparation of a saline solution, and the number of injections for different age groups until detumescence is achieved are listed as Supplemental Material (Supplement 2 [24,102,114]).

Repeated sympathomimetic injections with or without irrigation should always be undertaken prior to a decision for surgical intervention as they have a success rate of 43–81%. As outlined in a single center report, priapism in 71 adult patients was treated (among them 24 men with CML, mean duration of priapism 4.2 ± 1.4 days) with penile blood aspiration +/− phenylephrine irrigation. Operative shunt surgery had to be performed in 18 out of these 24 (75%) men [89]. In the cohort analyzed here, only one out of nine patients (Pat#2) with sufficient details reported on the management underwent operative shunt surgery after penile puncture and flushing had proven unsuccessful.

### 4.6. General Approaches to Leukostasis in CML

A diagnosis of pediatric CML in CP becomes highly probable if the triad of extremely high WBC (median WBC in pediatric CML: 240,000/µL) with associated normal or even elevated platelet counts (present in all patients with CML-CP) and an enlarged spleen (present in >80% of pediatric CML cases) is present [7,117]. In our cohort, the criteria for this triad were fulfilled by 15 out of all 16 (94%) patients. It must be stressed that in CML-BP-like in acute leukemia, the platelet count is usually low which was the case in Pat#1 exhibiting only 73.000 platelets per µL, while Pat#16 exhibited a platelet count of 152,000/µL which is just still in the lower normal rage.

Additional hints towards CML are given by the blood differential count showing a high number of immature granulocyte precursor cells (pathological “shift to the left”) while in contrast, acute leukemias exhibit a “hiatus leucemicus” (Latin, “leukemic gap”). Increased numbers of eosinophils and basophils typically are detected in more advanced stages of CML [54]. Thus, to identify the underlying hematological cause when encountering a boy with priapism, abdominal palpation and collecting blood for a WBC and blood film should be among the first diagnostic steps (Figure 3).

For unspecific treatment of blood hyperviscosity, hydration via administration of intravenous fluid is most important. To deplete the high number of white blood cells, leukapheresis (see below) should be considered if available without delay. Cytoreductive drugs, which should be started immediately once a diagnosis of CML is suspected, comprise high dose hydroxyurea (orally 50 mg/kg BW daily, maximum daily dose 4 g, in four divided doses per day) and cytarabine (intravenously 50 mg/sqm BS over 4 h daily). Also, the blood uric acid level should be checked and, if elevated, treated with allopurinol. Hydroxyurea and cytarabine will half the white cell count after 2–3 days until a safer level is achieved and can be continued until the diagnosis of CML is confirmed from bone marrow aspirates by detection of the Philadelphia chromosome and/or the BCR::ABL1-rearrangement [118]. At that point, tyrosine kinase inhibitors (TKIs) such as imatinib, nilotinib, and dasatinib licensed for targeted therapy in minors are the drugs of choice which have revolutionized the treatment of pediatric CML in the last two decades [7,15,68]. A detailed review of the efficacies and side effects of TKIs is not within the scope of this review. However, it should be stressed that the initial leukocyte halving time is not shorter than 8–14 days with TKI as a targeted treatment approach.

### 4.7. Leukapheresis and Exchange Transfusion

As chemotherapy requires time before becoming effective in resolving hyperleukocytosis, management of symptoms and signs of leukostasis may require temporary management by leukapheresis or exchange transfusion. Pros and cons of both procedures are outlined in more detail in Supplement 3 [19,49,50,64,119,120,121,122,123,124,125,126,127]. A decision to perform leukapheresis requires an apheresis team experienced to perform the procedure in children. Low patient weight (<10 kg), presence of coagulopathy including thrombocytopenia in patients with CML-BP, and risk for other metabolic or organ dysfunctions are factors to prefer manual blood exchange.

A single leukapheresis procedure can reduce the WBC count by 30–60% and case series have reported the successful use of therapeutic leukapheresis in combination with cytotoxic therapy to treat priapism [19,45,99,128,129]. A peripheral WBC of less than 100,000/µL has been reported as the goal, while some advocate that the patient’s symptoms will guide the adequacy of leukapheresis [19,45].

In the cohort analyzed here, five out of nine (55%) boys underwent leukapheresis after a penile puncture had been performed (Table 1, Case Vignette #2). Keeping in mind the small size of our cohort, this proportion is more than twice as many as in the, so far, largest series analyzing literature reporting on patients with priapism and CML where leukapheresis was performed in only 7/34 (21%) pediatric patients [90]. However, this rather low percentage might be biased by the rather extended time frame of 60 years (1960–2020) from which the cases were collected from the literature.

In the Japanese series, three of four boys with priapism underwent therapeutic aspiration and two of these underwent leukapheresis [51]. In another report on two CML patients (one boy with priapism, one girl with blurred vison due to leukostasis) leukapheresis was efficient and successful, resulting in lower numbers of circulating white cells and paralleled by resolution of clinical signs of leukostasis in both teenagers [130].

Performing manual whole blood exchange transfusion (MWBET) in children with leukemia is recommended in acute leukemia protocols to handle emergency scenario resulting from hyperleukocytosis [49,50,119,120]. However, experience is generally limited, and most pediatric hemato-oncologists and ICU staff are not adequately trained in this procedure. No detailed recommendations or guidelines on when and how to perform MWBET in patients with CML are available.

In consultation with transfusion services an isovolumetric exchange is usually performed through simultaneous infusion of reconstituted whole blood (packed red cells + fresh-frozen plasma to the Hct of the patient) via one venous access and removal of the patient’s leukocyte-rich blood via another venous canula [121]. Alternatively, MWBET is performed using one central venous line and employing a push–pull technique. MWBET replaces the patient’s leukemia-rich blood at a 1:1 volume ratio; therefore, the patient remains euvolemic, the anemia is not exacerbated, and metabolic abnormalities may be corrected as completed in as little as 2 h, but obviously the weight of the patient and the resulting volume exchanged affects this factor [131]. For more details the reader is kindly referred to Supplement 3.

### 4.8. Stepwise Approach to Manage a Pediatric CML Patient with Priapism

There are no standard treatment recommendations and no standardized approach to treat pediatric leukemic priapism. For adults, it is strongly recommended by the American Urological Association that systemic treatment of a hematological disease, such as CML, should not be undertaken as the only treatment for priapism [114]. Also, the European Guideline stresses the priority of an aggressive and stepwise management with the goal to achieve prompt penile detumescence [23].

Patients with priapism seeking telephonic advice should be encouraged as “first aid” measures to drink water (minimal volume 1% of the body weight) as an attempt to lower blood viscosity but to withdraw from food intake keeping in mind that anesthesia might be necessary within the next hours. As adrenergic measure, physical exercise (e.g., running-up staircases) has proven successful in patients with sickle cell disease and may be advocated also in cases of CML depending on the overall performance of a patient with leukemia [132]. In addition, urination and masturbation with ejaculation in teenagers may result in detumescence [24]. A cold bath or shower, or cold packs should only be recommended if sickle cell disease can be ruled out as cold compresses are contraindicated in sickle cell disease [133]. Applying cold packs is analgesic and, in addition, reduces tissue metabolism which might promote a cytoprotective effect and limit ischemic damage. These simple steps have occasionally been sufficient enough to produce detumescence [24]. However, all patients with a priapic episode lasting longer than two hours, should be examined as emergency cases by a physician for further assessment and “first aid” attempts should not postpone decisions to proceed to the next levels of treatment.

The first medical steps comprise evaluation of the underlying pathology including history taking, physical examination, and blood tests. The triad of splenomegaly plus hyperleukocytosis plus regular or elevated platelet counts make a diagnosis of CML highly probable. If available, a penile Doppler ultrasound is the imaging procedure of choice to differentiate low-flow from high-flow priapism.

If a Doppler ultrasound cannot be performed straight forwardly, the second step is penile puncture and aspiration of blood for a gas analysis (Figure 4). This procedure in children and teenagers should be performed under anesthesia, preferably using ketamine (Supplement 1). A dark red color of the blood and typical hypoxic values in the blood gas analysis will confirm a diagnosis of low-flow priapism. Following puncture using a butterfly needle and an attached three-way-tap, the blood is aspirated, and the corpora cavernosa flushed with saline until the blood color changes to light red thus reducing the cavernosal pressure.

If detumescence is not achieved at this point, as a third and next step, injection of sympatho-mimetic solution is required. This should be performed with monitoring of blood pressure and electrocardiogram. The required diluted solution as outlined in Supplement 2 should be prepared before aspiration.

If priapism persists at this point, a spongio-cavernous shunt (a distal shunt is preferred to a proximal shunt) is required as a fourth step. Listing details and pro and cons on these surgical procedures is far behind the scope of this article. The reader is kindly referred to references cited describing different shunt operations at pediatric age and outcome [24,27,93].

Following step 3 or step 4, systemic anti-leukemic therapy of CML with chemotherapy and leukapheresis or exchange transfusion should commence to avoid recurrence of priapism. The response to cytostatic drugs alone is usually prolonged over days to weeks and cases treated solely by this approach may represent the natural history of ischemic priapism rather than the drug effect [99,114]. Leukapheresis or exchange transfusion should be performed on the pediatric ICU because complication of leukostasis might also affect other organs (retina, inner ear, CNS, lung, and heart) [7].

In the series of articles and case reports analyzed by E. Ali and coworkers comprising 102 patients with CML and priapism diagnosed over a rather long period of 60 years (1960–2020), 34/102 patients were ≤18 years old [90]. In 14 out of 28 (50%) patients (6/34 patients no data reported), upfront cytostatic drug treatment was initiated. Half of the cohort (15/30 patients, 4/34 patients no data reported) underwent penile blood aspiration and cavernosal irrigation. Leukapheresis was performed in only 7/34 (20%) pediatric patients. A total of 14/34 (41%) patients required a shunt procedure. Also of note, any individual boy from these cohort may have undergone several out of the procedures indicated. Concerning the long-term outcome, no data were available in 21/34 (62%) patients. In the remaining 13/34 (38%) patients, erectile function was maintained in 8/13 (61%), but lost in 5/13 (39%) boys. These data underline the importance of cavernosal plus systemic treatment in a timely fashion [90].

Interestingly, M. Castagnetti and coworkers report managing three prepubertal boys (all aged 9 years) with CML and priapism without penile puncture but cytostatic drugs and intravenous fluids plus leukapheresis in two of them [129]. Detumescence was noticed late after 13 days in all three of them, but resolution of pain was observed immediately after leukapheresis. The authors postulate that erection without pain might be a sub-type of priapism not requiring any treatment since it would not be associated with any penile ischemia. None of the boys developed clinical evidence of penile fibrosis or erectile dysfunction after 4–8 years follow-up. This is in sharp contrast to the outcome in patients with sickle cell disease who do not have leukocytosis but represent a cohort where much larger experience is accumulated in pediatric patients than in the rare group of priapism in pediatric CML [39,90,134].

In the cohort described here, data on long-term outcome penile function were reported by 7 out of 15 (47%) of the patients. In most cases during the years-long treatment of CML, patients were transferred form pediatric departments to internal hematologist and lost to follow-up. With the limitation of a very small number of cases analyzed, it is good to learn that at least in all seven responders to the questionnaire, no erectile dysfunction was reported. As of note, prior to intervention, only two (Pat#1; Pat#5) of the seven boys had experienced a continuous priapic episode of maximally 12 h duration, while stuttering priapism lasting over a period from 24 h (Pat#1) to six weeks (Pat#3) or of unknown duration (Pat#10) was reported by the remaining five patients. However, without doubt, this cohort is too small and heterogenous to speculate whether or not long stuttering ischemic intervals are tolerated in children or adolescents before irreversible damage and fibrosis occurs.

When presenting with priapism, only with prompt diagnosis and standardized treatment may patients have a good prognosis for life and erectile function. Therapeutic measures require a multidisciplinary approach with pediatric oncologists, urologists, radiologists, apheresis experts, nursing, and psychology.

### 4.9. Psychological Problems

Studies from cohorts of patients with sickle cell disease have demonstrated a common failure in pubertal boys to disclose the priapism at its onset to their parents [135]. Typically, a teenager may feel embarrassed and uncertain about whether this experience is part of his normal sexual development. Prevention to disclose the problem frequently is associated with stuttering priapism. Teenagers and their parents often feel uncomfortable when discussing a boy’s sexual health [25,29]. This attitude may further delay diagnosis and adequate management [25]. Even when attending the emergency room, patients reported being embarrassed with the perception that it is a sexual issue, although the priapism without doubt had clearly reached the stage of being a medical emergency [135].
**Case vignette #3** **Fatal consequence of erectile dysfunction following priapism** In 1987, stuttering priapism over two weeks followed by a painful erection of 16 h occurred in a ten-year-old boy and was treated with a spongiocavernous shunt operation after penile blood aspiration and adrenaline injection had not resulted in detumescence. Underlying CML was treated by hydroxyurea for leukoreduction. Four months later after conditioning by busulfan plus cyclophosphamide, an allogeneic SCT was performed from his HLA-identical sister as bone marrow donor. The course post SCT was uneventful with low acute toxicity and no acute, but mild, chronic GvHD (dry, itching skin) reported. Erectile function was not assessed during routine follow-up visits. After finalizing school education successfully, the former patient started studying at a University of Applied Sciences to become a social welfare worker. Erectile dysfunction prohibited ordinary sexual intercourse and azoospermia was diagnosed due to busulfan-based conditioning. Both sequelae resulted in mental depression. When his girlfriend terminated their relationship after one year of courting, he committed suicide by hanging at the age of 25 years.

At its extreme, low-flow priapism lasting for longer than 24 h may result in partial or total impotence by erectile dysfunction. This physical disability can exert a large psychological impact on patients’ lives (Case Vignette #3). While there are guidelines produced by the American Urological Association on management of erectile dysfunction, individuals’ wellbeing may be reduced by intimacy avoidance, fear to attract or keep a partner, resulting in loneliness, loss of self-esteem, and despair [24,98,133,135].

## 5. Conclusions

Priapism can be the first manifestation of previously undetected CML. It is a very rare event in boys requiring urgent emergency therapy to avoid the risk of erectile dysfunction. It has not been investigated in a systematic fashion and due to its rarity, most publications are pediatric case reports and small case series. Recommendations for adult patients emphasize a combined approach to the management of priapism in CML. Adopted to pediatric patients and in line with a proposed pediatric clinical guideline for the management of various kinds of priapism in children, immediate local intracavernous therapy should be combined with cytoreductive measures such as leukapheresis, exchange transfusion, and cytotoxic therapy (hydroxycarbamide, low-dose cytarabine, TKI). Treatment of CML should not preclude or delay penile puncture to reduce the child’s priapism. Psychological sequelae may also be reduced with early intervention.

## Figures and Tables

**Table 1:** Types of pediatric leukemias and propensity for hyperleukocytosis and leukostasis.

Pediatric Leukemia Type	Cell Size	Cell Rigi- dity	Relative Propensity for Hyperleukocytosis (Defined as WBC > 10^9^/L)	Relative Propensity for Leukostasis *	Reference
			**low—moderate**	**moderate**	
Acute lymphoblastic leukemia (ALL)	**moderate**	**high**	at diagnosis in 74/611 (12%) pediatric patients at diagnosis in 84/634 (13%) pediatric patients	not indicated treated in 18/84 (21%) patients with hyperleukocytosis	[48] [49]
			**moderate**	**high**	
Acute myeloid leukemia (AML)	**large**	**high**	at diagnosis in 238/1251 (19%) pediatric patients at diagnosis in 18/143 (12%) pediatric patients	treated in 83/238 (25%) patients with hyperleukocytosis treated in 12/18 (66%) patients with hyperleukocytosis	[50] [49]
			**high**	**low**	
Chronic myeloid leukemia (CML)	**variable**	**low**	at diagnosis in 32/40 (80%) pediatric patients at diagnosis in 162/256 (63%) pediatric patients	treated in “only a few patients” with hyperleukocytosis treated in 23/162 (14%) patients with hyperleukocytosis	[9] [51]

* In the references cited, leukostasis was defined clinically and recorded if interventions such as exchange transfusion or leukapheresis procedures were performed. These procedures might also have been applied prophylactically based solely on the WBC in selected cases of acute leukemias.

**Table 2:** Patients‘ characteristics, duration of priapism, medical intervention, and outcome.

Pat No/Country	Age at Dx [yrs]/ Date of Dx [mo/year]	CML Phase	Spleen Size [Below c. m.]	WBC [/µL]	Hb [g/dL]	Platelets [/µL]	Duration of Priapism Prior to Dx	Intervention Regarding Priapism	Late Outcome Regarding Penile Function	Treatment and Outcome of CML
1/GER	4 05/2008	BP	8 cm	899,000	5.1	73,000	24 h, stuttering	exchange trans- fusion	normal at age 20 yrs	ALL induction + IMA, SCT 07/2008, last FU 09/2019, PCR neg.
2/GER	7 10/2006	CP	17 cm	508,000	8.4	537,000	<12 h continuous erection	penile puncture, adrenaline injection, spongiocavernous shunt, leukapheresis 3×	unknown	IMA, MR3 05/2005, MR4 08/2006, switch DASA 10/2010, switch NILO 06/2012
3/GER	7 06/2010	CP	7 cm	635,000	4.9	186,000	>6 weeks, stuttering	penile puncture, leukapheresis 2×, low dose ARA-C	normal at age 20 yrs	IMA, switch to DASA 09/2016, MR4 1/2016, DMR 06/2016, last FU 10/2020
4/POL	9 01/2013	AP	6 cm	483,000	9.2	403,000	5–7 days, stuttering	hydration, HUS	normal at age 20 yrs	HUS 1 week, IMA, MR3 10/2014, last FU 05/2023 on IMA, DMR
5/GER	10 07/2014	CP	12 cm	629,000	6.4	361,000	11 h continuous erection	leukapheresis 2×, HUS 17 d, low dose ARA-C	normal at age 18 yrs	IMA, MR3 07/2015, ongoing IMA, DMR 01/2016, last FU 07/18
6/TUR	11 05/2006	CP	4 cm	459,000	7.6	655,000	unknown	unknown, *a	unknown	HUS 1 mo, IMA 06/2006, no data on PCR, lost to FU 08/2013
7/FRA	11 01/2007	CP	12 cm	368,510	7.1	826,000	≥12 h continuous erection	unknown	unknown	IMA, MR3 04/2008, bad compliance 2/2012, MR4 6/2014, DASA 09/2016, DMR 12/2019
8/FRA	13 06/2000	CP	4 cm	339,000	6.7	1,130,000	several days stuttering	penile puncture, adrenaline inject-tion heparin, urokinase, HUS	not applic- able, early death	Chemotherapy (FRALLE 2000), SCT cord blood UD 2/2001, death 07/2001 pneumonitis + infections
9/FRA	14 01/2008	CP	12 cm	407,840	8.0	373,000	several days stuttering, recurrence 2 days after diagnosis of CML	HUS at diagnosis of CML, at recurrence: penile puncture adrenaline injection, leukapheresis	unknown	IMA, secretly stopped therapy for 1 year 2010–2011, HD-IMA not tolerated, cardiac tox., switch to DASA 05/2011, MR3 10/2012, MR4 04/2015, DMR 06/2016, stopping attempt failed 2019, regained MR3 02/2020
10/GER	14 08/2014	CP	2 cm	450,000	9.1	265,000	>3 months, stuttering	exchange trans- fusion, low dose ARA-C	normal at age 22 yrs	IMA, switch to DASA 09/2015, MR3 05/2016, last FU 02/2023
11/TUR	15 11/2005	AP	15 cm	657,270	6.5	434,000	unknown	unknown	unknown	IFN 2 weeks, IMA, SCT sib. donor 05/2006, PCR neg. post SCT
12/GER	15 09/2018	CP	14 cm	350,240	8.5	980,000	11 h continuous erection	penile puncture adrenaline inject-ion, leukapheresis, HUS	normal at age 20 yrs	IMA, switch to DASA 10/2019, no stable MR3, last FU 08/2021
13/POL	16 09/2007	CP	10 cm	481,170	8.1	253,000	<2 weeks stuttering	penile puncture, leukapheresis HUS	normal at age 25 yrs	HUS 1 mo, IMA 3 mo, HUS again 5 mo (logistic problems with IMA supply), IMA since 04/2008, MR3 09/2011, stopped treatment 07/2017 due to unknown (personal) reasons, died in 08/2018 due to CNS bleeding in the course of CML-BP
14/GER	17 01/2008	CP	3 cm	236,700	9.7	386,000	unknown	unknown	unknown	IMA, MR3 11/2009, last FU 08/2011
15/BRA	17 06/2012	CP	14 cm	355,470	14.0	427,000	unknown	unknown	unknown	IMA, MR3 12/2015, MR4 4/2016, DMR 3/2019
16/FRA	17 05/2018	BP	4 cm	481,000	9.8	152,000	unknown	heparine + prednisolone	unknown	ESPHAL including IMA, MR3 11/2018, SCT sib. donor 11/2018, relapse 02/2019, IMA post SCT, MR4 12/2019

* a penile Doppler ultrasound showed thrombosis in corpora cavernosa. **Abbreviations:** ALL = acute lymphoblastic leukemia, AP = accelerated phase of CML, ARA-C = cytarabine, BP = blastic phase of CML, BRA = Brazil, c. m. = costal margin, CML = chronic myeloid leukemia, CP = chronic phase of CML, DASA = dasatinib, DMR = deep molecular response with a BCR::ABL1 transcript ratio of ≤0.0032%, Dx = diagnosis, ESPHAL = European Study for Philadelphia-pos. Acute Lymphoblastic Leukemia, FRA = France, FRALLE = French Acute Lymphoblastic Leukemia in childhood, FU = follow-up, GER = Germany, h = hours, IFN = interferon alpha 2, HD-IMA = high-dose imatinib (600 mg), HUS = hydroxyurea, IMA = imatinib, mo = month, MR3= BCR::ABL1 transcript ratio of 0.1%, MR4 = BCR::ABL1 transcript ratio of 0.01%, neg = negative, NILO = nilotinib, PCR = quantitative reverse transcript polymerase chain reaction for monitoring of the BCR::ABL1 transcript; POL = Poland, SCT = allogeneic stem cell transplantation, sib = sibling, tox = toxicity, TUR = Turkey, yrs = years, UD = unrelated donor, WBC = white cell blood count.

## Data Availability

The data presented in this study are not publicly available due to personal data restrictions but are available from the corresponding author on request. This excludes any individual personal/clinical data of the individuals, which would endanger their anonymity.

## Supplementary Materials

The following supporting information can be downloaded at https://www.mdpi.com/article/10.3390/jcm12144776/s1: Supplement 1 Anesthesia in particular ketamine; Supplement 2 Preparation of sympathomimetic solutions, including Table S1; Supplement 3 Mechanical leukocyte-reductive measures.
