# Supplementary material for: Priapism at Diagnosis of Pediatric Chronic Myeloid Leukemia: Data Derived from a Large Cohort of Children and Teenagers and a Narrative Review on Priapism Management
Source: J Clin Med. 2023 Jul 19;12(14):4776. doi: 10.3390/jcm12144776 (PMC10380995; doi:10.3390/jcm12144776)
Supplement: Supplementary file 1 [file jcm-12-04776-s001.zip › jcm-2455947-supplementary.docx]

**Supplemental Material**

**Supplement 1**

**Anesthesia, in particular ketamine**

Pain management for children undergoing painful medical procedures is an important pediatric problem [104]. In achieving penile detumescence without delay, the aim is to avoid erectile dysfunction as sequela of priapism, and the length of time to initiate necessary procedures should be minimized while in parallel patient’s discomfort from pain and distress must be reduced. To achieve this goal, regimes selected vary widely and include sedation, general anesthesia, and more time-consuming psychological therapies such as distraction, hypnotherapy, and relaxation techniques.

For general anesthesia, ketamine, in particular, may offer therapeutic benefit. Induction of anesthesia with 2 mg/kg bodyweight (BW) of ketamine usually produces 15 to 30 minutes of unconsciousness within seconds. Ketamine may also be administered rectally at 10 mg/kg BW, orally at 6 to 10 mg/kg BW, or intranasally at 3 to 6 mg/kg BW to induce anesthesia [105]. Ketamine has established effectiveness in resolving priapism in the adult and pediatric population [106-109]. It does not significantly affect blood pressure and/or cardiac output, but exerts alpha- and beta agonistic effects on adrenergic receptors of the peripheral vasculature which possibly explains its effect in returning penile blood flow to normal. Ketamine further acts as an agonist on the opioid sigma receptor, as antagonist of the cholinergic receptors, and antagonist of reuptake pumps for catecholamines [110]. These effects cause dissociation between the thalamocortical and limbic systems, which coordinates central sensory and visceral inputs involved in erectile function [111]. In addition, dissociation on the limbic system produces anesthesia, analgesia, suppression of fear and anxiety, and amnesia.

When selecting the optimal modality of anesthesia, the risks of lasting psychological damage, aspiration complications, anesthetic risk, and loss of time increasing the probability of erectile dysfunction must be well balanced. Ketamine may be a preferred anesthetic drug for the necessary procedures when treating pediatric priapism and may even obviate the need of penile blood aspiration [107].

**References Supplement 1 (****numbered according to the main text of the manuscript)**

104. Harvey AJ, Morton NS. Management of procedural pain in children. Archives of Disease in Childhood - Education and Practice 2007;92:ep20-ep26.

105. Simonini A, Brogi E, Cascella M, Vittori A. Advantages of Ketamine in Pediatric Anesthesia. Open Med. 2022;17:1134–1147. doi: 10.1515/med-2022-0509.

106. Zipper R, Younger A, Tipton T, Jackson B, Prasad M, Hayden G, Stec A. Ischemic priapism in pediatric patients: Spontaneous detumescence with ketamine sedation. J Pediatr Urol. 2018 Oct;14(5):465-466.

doi: 10.1016/j.jpurol.2018.05.005. Epub 2018 Jun 7. PMID: 29936034.

107. Park DB, Hayden GE. Ketamine Saves the Day: Priapism in a Pediatric Psychiatric Patient. Pediatr Emerg Care. 2015 Jul;31(7):508-10. doi: 10.1097/PEC.0000000000000485. PMID: 26148100.

108. Villalonga A, Beltran J, Gomar C, Nalda MA. Ketamine for treatment of priapism. Anesth Analg 1985;64:1033-1034.

109. Benzon HT, Leventhal JB, Ovassapian A. Ketamine treatment of penile erection in the operating room. Anesth Analg 1983;62:457-458.

110 Bergman SA. Ketamine: review of its pharmacology and its use in pediatric anesthesia. Anesth Prog. 1999;46:10–20

111. Gale AS. Ketamine prevention of penile turgescence. JAMA. 1972; 219:1629.

**Supplement 2**

**Preparation of sympathomimetic solutions**

Until detumescence is achieved, phenylephrine should be administered at a concentration of 200 µg/ml diluted in saline 0.9% and injected in 0.5 ml doses (100 µg) every 5–10 min in children aged over 11 years. The cumulative maximal dose of 1 mg (= 1000 µg) should not be overridden (Table S5). Lower concentrations should be used in children and patients with cardiovascular disease. Children as young as 3 years have received epinephrine (adrenaline) for irrigation in a dilute solution [24,102,114].

**Table S1:** Preparation of sympathomimetic solutions for penile intracorporal injection (Caveat: Unlicensed indication and route of administration. Adopted from [24]. For details see text and reference.

| **Drug** | **Preparation** | **Concentration** | **Age and aliquot** | **Further doses** |
| --- | --- | --- | --- | --- |
| Phenylephrine | 10 mg/ml (1%) | 0.1 ml + 4.9 ml 0.9% saline (200 µg/ml) | ≥11 yrs: 0.5 ml | ≤10 at 5 –10 min (≤1 mg) |
| Epinephrine  (adrenaline) | 1 in 10 000  (100 µg/ml) | 1 ml + 99 ml 0.9% saline  1 in 1 000 000 or 1 µg/ml) | ≥11 yrs: 15 ml  3 – 11 yrs: 10 ml | ≤4 at 10 min |
| Etilefrine | 10 mg/ml (1%) | – | 3 – 18 yrs: 0.5 mg/ml | ≤2 at 10 min |

**References S2 (numbered according to the main text of the manuscript)**

24. Donaldson JF, Rees RW, Steinbrecher HA. Priapism in children: a comprehensive review and clinical guideline.

J Pediatr Urol 2014 Feb;10(1):11-24. doi: 10.1016/j.jpurol.2013.07.024. Epub 2013 Sep 8. PMID: 24135215.

102. Mantadakis E, Ewalt DH, Cavender JD, Rogers ZR, Buchanan GR. Outpatient penile aspiration and epinephrine irrigation for young patients with sickle cell anemia and prolonged priapism. Blood 2000;95:78e82.

114. Montague DK, Jarow J, Broderick GA, Dmochowski RR, Heaton JP, Lue TF, Nehra A, Sharlip ID; Members of the Erectile Dysfunction Guideline Update Panel; Americal Urological Association. American Urological Association guideline on the management of priapism. J Urol. 2003 Oct;170(4 Pt 1):1318-24. doi: 10.1097/01.ju.0000087608.07371.ca. PMID: 14501756.

**Supplement 3**

**Mechanical leukocyte-reductive measures**

**Leukapheresis**

As a single leukapheresis procedure can reduce the WBC count by 30–60%, repetitive daily treatments may be necessary until the number of white blood cells is lowered; however, at pediatric age, a single procedure may control leukostasis symptoms in most boys [19]. The initial leukapheresis procedure may also relief penile pain immediately while other signs of prolonged priapism such as penile edema was not reduced significantly [122]. Potential disadvantages of leukapheresis comprise the limited availability of trained staff with expertise, necessity of sophisticated technical equipment, and operational capability during emergency hours at smaller hospitals.

Leukapheresis results in a significant extra corporal shift of net blood volume requiring careful fluid balance to maintain circulatory euvolemia [123]. As pediatric patients with CML typically present at diagnosis with anemia [64], care must be taken not to worsen the red cell deficiency and no concerns to prime the apheresis machine with donor blood should be relevant. In addition, the caliber of venous access to ascertain the minimally required blood flow towards the separator and the risks of necessary anticoagulation in conjunction with the citrate toxicity arising from ACD-A („Acid-Citrate-Dextrose-Adenine”) used as packed red cell stabilizer, requiring frequent calcium monitoring and supplementation must be well balanced, especially in smaller children [121]. In patients with CML-BP-lymphoid receiving steroid therapy, rapid tumor cell lysis might result in hypocalcemia, hyperkaliemia, and other electrolyte derangements, thus requiring frequent monitoring.

For cases of malignant hyperleukocytosis, guidelines on apheresis give recommendations for adults with AML, while in pediatric AML, leukapheresis is recommended with restraints [50,124,125]. Following recommendations for adults, its role in ALL is unclear, and for CML, no recommendation is given [124]. The latter—among other reasons—may contribute to the management of priapism without leukapheresis. However, due to the lack of randomized studies, the role of leukapheresis in the management of complications of CML is still uncertain. Concerning priapism, leukapheresis must target at a rapid improvement of penile tissue perfusion and thus remains an emergency medicine multidisciplinary approach, including expertise from hematology, urology, and transfusion medicine departments.

**Exchange transfusion**

Prior to the implementation of Anti-Rh-D immunization prophylaxis in Rhesus blood group negative pregnant woman, manual whole blood exchange transfusion (MWBET) in newborns with severe hyperbilirubinemia from hemolytic disease used to be a frequently performed procedure. Due to the impressively high success rate of the prophylaxis, clinical experience with MWBE in newborns has been declining. While performing MWBE in children with leukemic hyperleukocytosis is recommended in acute leukemia protocols to handle emergency scenarios [50,49,119,120], due to the low frequency of leukostasis, experience in pediatric leukemia generally is limited. Decisions taken are based on individual experience and generally favor MWBET over leukapheresis in smaller children (explicitly if <10 kg BW) due to smaller associated technical and complication risks [50,121,125].

Using either two venous cannulas for removing the patient’s blood and infusion of reconstituted whole blood or performing MWBET via one central venous line and employing a push–pull technique an isovolumetric exchange is usually performed. The recommended volumes not to be exceeded in neonates may also be appropriate in older children and comprise an infusion/removal rate of 3 mL/kg/min for the isovolumetric exchange technique or 5 mL/kg over a 2-4-minute cycle for the push–pull technique [126]. MWBET targeting at a patient’s double total blood volume exchange is more effective than a single-volume exchange in leukemic cell removal. Compared to leukapheresis which can reduce the total leukocyte count by 30% to 60%, MWBET can remove up to 85% of leukocytes [124]. In very young children the time required for the procedure can safely be completed in as little as 2 hours but, obviously, the weight of the patient and the resulting volume exchanged affects this factor [123].

Based on a bodily total blood volume of approximately 80 ml/kg, high numbers of red cells units may be required for older children. In the series reported here, two boys, 4 and 14 years old, respectively, were treated by exchange transfusion, with the 14-year-old boy (Pat#10) receiving a total volume of 20 L of donor blood. Ideally, fresh (<1 week old) leukoreduced, irradiated units of ABO/D compatible red cells should be reconstituted with fresh frozen plasma to a final Hct equivalent to the Hct of the patient [121]. This should minimize any risk of hyperviscosity resulting from hyperleukocytosis and in conjunction with possible thombocytosis. Like in leukapheresis, monitoring of serum calcium is mandatory in MWBET since citrate exposure occurs with the reconstituted whole blood. Of note, in patients with CML-BP and associated thrombocytopenia, it is necessary to monitor platelets as the replacement product contains RBCs and FFP only [127].

**References Supplement 3 (numbered according to the main text of the manuscript)**

19. Korkmaz S. The management of hyperleukocytosis in 2017: Do we still need leukapheresis? Transfus Apher Sci. 2018 Feb;57(1):4-7. doi: 10.1016/j.transci.2018.02.006. Epub 2018 Feb 20. PMID: 29477941.

49. Abla O, Angelini P, Di Giuseppe G, Kanani MF, Lau W, Hitzler J, Sung L, Naqvi A. Early Complications of Hyperleukocytosis and Leukapheresis in Childhood Acute Leukemias. J Pediatr Hematol Oncol. 2016 Mar;38(2):111-7.

doi: 10.1097/MPH.0000000000000490. PMID: 26794706.

50. Creutzig U, Rössig C, Dworzak M, Stary J, von Stackelberg A, Wössmann W, Zimmermann M, Reinhardt D.

Exchange Transfusion and Leukapheresis in Pediatric Patients with AML With High Risk of Early Death by Bleeding and Leukostasis. Pediatr Blood Cancer. 2016 Apr;63(4):640-5. doi: 10.1002/pbc.25855. Epub 2015 Dec 16. PMID: 26670831.

64. Delehaye F, Rouger J, Brossier D, Suttorp M, Güneş AM, Sedlacek P, Versluys B, Li CK, Kalwak K, Lausen B, Srdjana C, Dworzak M, Hraskova A, De Moerloose B, Roula F, Briant A, Parienti JJ, Millot F. Prevalence of anemia at diagnosis of pediatric chronic myeloid leukemia and prognostic impact on the disease course. Ann Hematol. 2022 Nov 12.

doi: 10.1007/s00277-022-05024-1. Epub ahead of print. PMID: 36370190.

119. Haase R, Merkel N, Diwan O, Elsner K, Kramm CM. Leukapheresis and exchange transfusion in children with acute leukemia and hyperleukocytosis. A single center experience. Klin Padiatr. 2009 Nov-Dec;221(6):374-8.

doi: 10.1055/s-0029-1239533. Epub 2009 Nov 4. PMID: 19890790.

120. Zeller B, Glosli H, Forestier E, Ha SY, Jahnukainen K, Jónsson ÓG, Lausen B, Palle J, Hasle H, Abrahamsson J; NOPHO AML working group. Hyperleucocytosis in paediatric acute myeloid leukaemia - the challenge of white blood cell counts above 200 × 109 /l. The NOPHO experience 1984-2014. Br J Haematol. 2017 Aug;178(3):448-456.

doi: 10.1111/bjh.14692. Epub 2017 May 25. PMID: 28542715

121. Runco DV, Josephson CD, Raikar SS, Goldsmith KC, Lew G, Pauly M, Fasano RM. Hyperleukocytosis in infant acute leukemia: a role for manual exchange transfusion for leukoreduction. Transfusion. 2018 May;58(5):1149-1156.

doi: 10.1111/trf.14512. Epub 2018 Feb 4. PMID: 29399859; PMCID: PMC5912979.

131. Jain R, Bansal D, Marwaha RK. Hyperleukocytosis: emergency management. Indian J Pediatr. 2013 Feb;80(2):144-8. doi: 10.1007/s12098-012-0917-3. Epub 2012 Nov 24. PMID: 23180411.

122. Noushad S, Sahoo D, Basavarajegowda A, Toora E, Paudel P. Effect of leukapheresis on pain reduction in leukemic priapism. Asian J Transfus Sci. 2022 Jan-Jun;16(1):124-127. doi: 10.4103/ajts.ajts_160_21. Epub 2022 Jul 30. PMID: 36199404; PMCID: PMC9528545.

123. McLeod B. Pediatric therapeutic apheresis. In: McLeod, B., editor. Therapeutic apheresis: a physician’s handbook. Bethesda: AABB; 2005. p.135-62.

124. Schwartz J, Padmanabhan A, Aqui N, Balogun RA, Connelly-Smith L, Delaney M, Dunbar NM, Witt V, Wu Y, Shaz BH.

Guidelines on the Use of Therapeutic Apheresis in Clinical Practice-Evidence-Based Approach from the Writing Committee of the American Society for Apheresis: The Seventh Special Issue. J Clin Apher. 2016 Jun;31(3):149-62.

doi: 10.1002/jca.21470. PMID: 27322218.

125. Oberoi S, Lehrnbecher T, Phillips B, Hitzler J, Ethier MC, Beyene J, Sung L. Leukapheresis and low-dose chemotherapy do not reduce early mortality in acute myeloid leukemia hyperleukocytosis: a systematic review and meta-analysis. Leuk Res. 2014 Apr;38(4):460-8. doi: 10.1016/j.leukres.2014.01.004. Epub 2014 Jan 10. PMID: 24472688.

126 Ramasethu, J. Exchange transfusion. In: MacDonald, MR., Jayashree, R., editors. Atlas of procedures in neonatology.

Philadelphia: Lippincott Williams & Wilkins; 2013. p. 315-23.

127. Bunin NJ, Kunkel K, Callihan TR. Cytoreductive procedures in the early management in cases of leukemia and hyperleukocytosis in children. Med Pediatr Oncol. 1987;15(5):232-5. doi: 10.1002/mpo.2950150503. PMID: 3477680.
